# Investigation on the corrosive effect of NH_3_ during InGaN/GaN multi-quantum well growth in light emitting diodes

**DOI:** 10.1038/srep44850

**Published:** 2017-03-21

**Authors:** J. Yang, D. G. Zhao, D. S. Jiang, P. Chen, J. J. Zhu, Z. S. Liu, W. Liu, X. Li, F. Liang, S. T. Liu, L. Q. Zhang, H. Yang

**Affiliations:** 1 State Key Laboratory on Integrated Optoelectronics, Institute of Semiconductors, Chinese Academy of Sciences, PO Box 912, Beijing 100083, People’s Republic of China; 2 School of Electronic, Electrical and Communication Engineering, University of Chinese Academy of Sciences, Beijing 100049, China; 3 Suzhou Institute of Nano-tech and Nano-bionics, Chinese Academy of Sciences, Suzhou 215123, People’s Republic of China

## Abstract

Three series of samples with different NH_3_ flow rate are grown and the optical and structural properties are investigated. It is found that apart from a positive effect on keeping a high partial pressure of nitrogen to enhance indium incorporation, NH_3_ may also play a negative effect on indium incorporation during InGaN growth. Especially, when temperature is relatively high, the hydrogen generated from the dissociation of NH_3_ may suppress the chemical reaction which produces InN, leading to a reduced indium incorporation efficiency during the InGaN layer growth.

Considerable attention has been focused on InGaN-based light emitting devices during past few years due to their promising applications in solid-state lighting and laser display. The key issue for realizing InGaN light emitting devices is the preparation of high quality InGaN/GaN multi-quantum wells (MQWs) by metal-organic chemical vapor deposition (MOCVD)^1^,^2^,^3^,^4^. However, it has been proved to be more difficult than the GaN layer growth^5^. First, the indium incorporation is sensitive to the growth conditions, such as growth temperature, growth rate, V/III ratio^6^, and the hydrogen flow rate^7^. In addition, the growth temperature of InGaN should be much lower than GaN due to the low dissociation temperature of InN^8^. In fact, the growth of InGaN epilayer has to be performed at temperature range of 650–850 °C. In this case, the cracking of ammonia becomes less efficient due to the high kinetic barrier for breaking N–H bonds. The insufficient N precursor leads to an unexpected dissociation of the In–N bond. Thus, the indium content in InGaN layer will be suppressed even when indium metal droplets have formed on InGaN growing surface^9^. To increase the indium content and prevent the indium droplets from formation on the growing surface, extremely high V/III ratio is often used during InGaN growth in the past few years^10^. However, in this work, it is found that a negative effect of high NH_3_ flux on indium incorporation, i.e., a corrosive effect of NH_3_ for InGaN, should also be considered, which may lead to a lower indium content and lower emission intensity for InGaN/GaN MQWs. Now, we will focus on the variation of optical and structural properties of InGaN/GaN MQWs with the increase of NH_3_ flow rate during MOCVD growth and examine the corrosive effect of NH_3_ in detail.

## Experiments

Ten InGaN/GaN MQW samples were grown by an AIXTRON 3 × 2 in. close-coupled showerhead reactor on c-plane sapphire substrate. The structure of samples consisted of a 2-μm thick Si-doped n-type GaN layer (*n* = 3 × 10^18^ cm^−3^) grown at 1040 °C, a 3-period unintentionally doped InGaN/GaN MQW active region, and a 130-nm thick Mg-doped p-type GaN layer grown at 950 °C. For the growth of InGaN well layers, the Triethylgallium, trimethylindium flow rate and the growth time were kept as constants for all samples, but other conditions may be changed. Following the growth of each InGaN well layer, a GaN barrier layer was grown at the same temperature as InGaN QW layer. According to the growth temperature of InGaN/GaN MQWs, the 10 samples can be divided into 2 series, i.e., series I, including samples A, B, C, D, and E, the growth temperature is 720 °C and series II (samples F, G, H, I, and J), their growth temperature is 760 °C, higher than those samples of series I. Among each series of samples, the NH_3_ flow rate is changed during InGaN/GaN MQW growth. They are 0.75, 1.5, 3, 4.5, and 6 slm for samples A, B, C, D and E, and 3, 4.5, 6, 7.5, and 9 slm for samples F, G, H, I, and J, respectively. The detailed growth conditions for these samples are listed in Table 1 In order to check the indium content in InGaN/GaN MQWs accurately, an additional series III of InGaN films (samples T1 and T2) are grown and their growth temperature and NH_3_ flow rate are the same as those of InGaN QWs in samples F and J, respectively, but the layer thickness is much larger. After the epilayer growth, the samples were characterized by high resolution x-ray diffraction (HRXRD), temperature-dependent photoluminescence (PL) and electroluminescence (EL) spectroscopy. HRXRD was performed employing a Cu *K*α1 line of wavelength *λ* = 0.154 nm, temperature-dependent PL spectra from 10 to 300 K were recorded using a 325 nm He-Cd continuous wave laser with an emission power of 3 mW, and EL spectra are measured under a fixed injection current of 10 mA.

## Results

**Figure 1:**
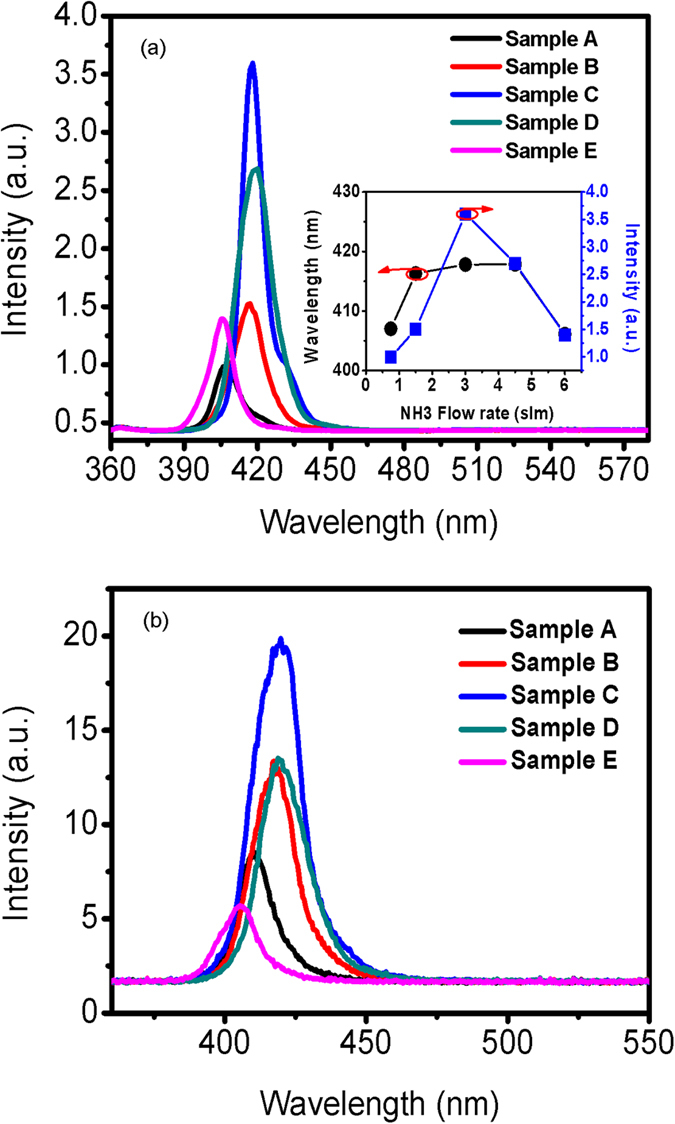
Room temperature photoluminescence (PL) and electroluminescence (EL) spectra of samples A, B, C, D and E. The inset in Fig. 1 (**a**) is the dependences of PL peak wavelength and peak intensity on the NH_3_ flow rate during InGaN/GaN MQW growth.

Room-temperature (RT) PL and EL spectra of samples A, B, C, D, and E are measured and shown in Fig. 1. The dependences of PL peak wavelength and intensity on NH_3_ flow rate are shown in the inset of Fig. 1(a). It can be seen from PL spectra that sample C (NH_3_ flow rate is 3 slm) has the strongest luminescence intensity in these samples. When the NH_3_ flow rate decreases from 0.75 to 3 slm during InGaN/GaN MQW growth, both of the peak wavelength and intensity increase. However, when it further increases from 3 to 6 slm, the emission peak wavelength and the peak intensity decrease. It suggests that the indium content or the thickness of InGaN decrease when the NH_3_ partial pressure (defined as NH_3_ flow rate/total flow rate times reactor pressure) is too large or too small during the InGaN layer growth.

**Figure 2:**
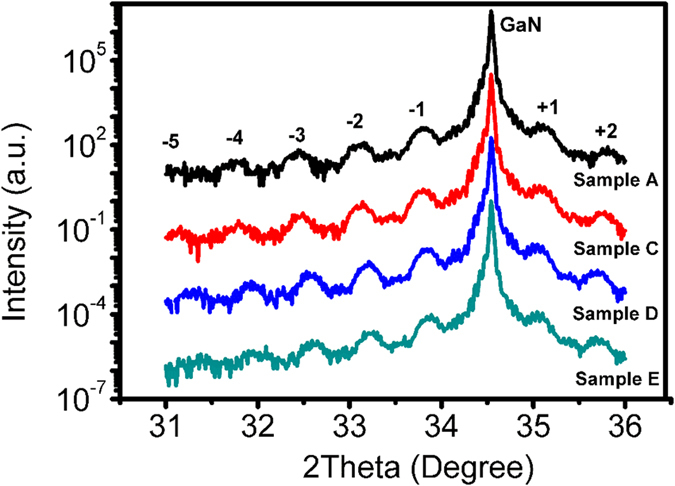
XRD ω-2θ curves on GaN (0002) plan for samples A, C, D and E.

In order to find the reason for a decrease in peak wavelength and peak intensity when NH_3_ partial pressure is too large or too small during InGaN/GaN MQW growth, XRD Omega-2theta curves of samples A, C, D and E are measured to check the indium content, period thickness and interface quality of InGaN/GaN MQWs. The Omega-2theta curves of (0002) plan are shown in Fig. 2. It is noted that in addition to the peaks at 34.56° originating from the underlying n-GaN layers, many high order satellite peaks arising from the periodicity of the MQWs can be clearly observed for all samples, indicating that a fine QW/QB periodic structure and an abrupt QW/QB interface are formed in these samples. It shows that, different from previous report^11^, the growth mode transition from step-flow growth to 2D island nucleation does not occur even when the NH_3_ flow rate decreases as low as 0.75 slm during the growth of our samples. In addition, the period thickness of MQWs and indium content of InGaN QWs are calculated based on the angular distance of the adjacent satellite peaks and the angular position of 0-th order peak of InGaN/GaN MQWs. The results are listed in Table 1. It can be seen that the period thickness is nearly the same for all these samples, but the indium content of InGaN well layers in samples A and E are lower than that in samples C and D, indicating that the indium incorporation efficiency is reduced when the NH_3_ partial pressure is too small or too larger. The XRD result agrees well with the result of peak wavelength shift obtained from PL measurement. The decrease of indium content in InGaN QWs when the NH_3_ partial pressure is too large or too small suggests that NH_3_ may play both positive and negative effects, i.e. both protective effect and corrosive effect, on the In incorporation into InGaN QWs. They are competitive to each other. When the NH_3_ partial pressure is too small, the amount of N precursor is insufficient, which leads to the dissociation of the In–N bond. In this case, NH_3_ flow rate should be increased to protect InGaN from decomposition. However, if the NH_3_ partial pressure is too large, the corrosive effect of NH_3_ may in turn play a leading role, resulting in a decrease of indium content of InGaN QWs.

In addition, to check the dislocation density in these samples, omega rocking curves are measured. The full width at half maximums (FWHMs) obtained for all samples are nearly the same, i. e, 300 and 350 arcsec for (0002) and (10–12), respectively, indicating that the dislocation density for all these samples is nearly the same. Thus we are aware that the dislocation density is apparently not the reason for the decrease of the emission intensity when the NH_3_ partial pressure is too small or too large.

**Figure 3:**
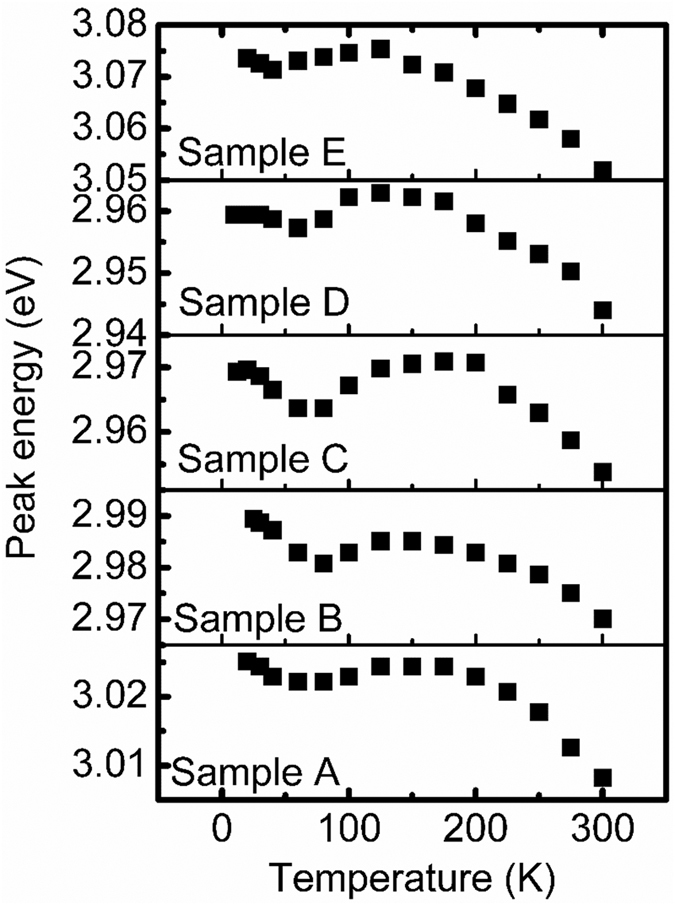
The variation of peak energy with temperature for samples A, B, C, D and E.

Apart from the dislocations, the localized luminescence centers in InGaN QWs may also play an important role on emission intensity of InGaN/GaN MQWs. Therefore, temperature-dependent PL spectra from *T* = 10 to 300 K for samples A, B, C, D and E are measured to study the localization luminescence centers in InGaN QWs. The dependencies of peak energy with temperature for samples A, B, C, D and E are shown in Fig. 3. It is found that an ‘S-shape’ temperature dependence of PL peak energy exists for all samples. The ‘S-shape’ behavior is an evidence of the presence of localized luminescence centers^12^,^13^, and they are often induced by the fluctuation of indium composition and/or well layer thickness of InGaN/GaN QWs^14^. To analyze the localization effect of different samples quantitatively, the dependences of peak energy on temperature are fitted based on the band tail model suggested by Eliseev *et al*^12^, a parameter σ is obtained and listed in Table 2. The value of *σ* gives an estimate to the degree of localization effect of samples^15^. The larger the *σ* value, the stronger the localization effect. In addition, the blueshift energy (Δ*E*) of PL peak appearing during the rise of temperature is also a parameter used to estimate the depth of localization states as the blueshift is caused by the escape of carriers from localization states. As shown in Table 2, the values of Δ*E* and σ for sample C are larger than those for other samples, indicating that the localization states in sample C are deeper, and the corresponding localization effect in it is stronger. According to PL and XRD measurements, it is assumed to be mainly attributed to the relatively higher indium content in InGaN QWs of sample C than in other samples.

On the other hand, the internal quantum efficiency (IQE) of all samples are estimated based on a standard method in which the RT integrated intensity is divided by integrated intensity at 10 K. The result is also listed in Table 2. It can be seen that even though the dislocation density of sample C is nearly the same as others, its IQE is much larger than those of samples A and E (i.e. the two samples grown with too small and too large NH_3_ partial pressure during InGaN/GaN MQW growth). Based on the discussion mentioned above, the localization states of samples C is deeper than others. We are aware that these localization states are the efficient luminescence centers in InGaN/GaN QWs, and they are able to help with preventing carriers from moving into the areas rich of dislocations to recombine non-radiatively there^16^,^17^. Thus, the higher IQE and higher RT PL intensity of sample C should be attributed to the existence of deeper localization states in InGaN/GaN MQWs. It means that by using a proper NH_3_ flow rate during InGaN growth to obtain relatively deep localization states in MQWs can contribute to an improvement of the emission efficiency for short wavelength light emitting devices.

Now we will discuss the question why using a too large NH_3_ partial pressure during InGaN growth leads to a relatively low indium content in InGaN layer ? It is known that InGaN is generated by the reactions of NH_3_, TMIn and TEGa, and H_2_ is an intermediate product of these reactions. However, additional dissociation of NH_3_ into N_2_ and H_2_ also occur at the same time by the reaction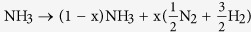, where the x is the fraction of dissociated NH_3_. It is enhanced when the reactor temperature increases^18^,^19^. In this case, the amount of hydrogen generated from the dissociation of NH_3_ will increase which plays a negative role in the indium incorporation as a “corrosive effect”. Actually, the InN percentage in MOCVD grown InGaN significantly influenced by the amount of hydrogen has been reported by many groups^20^,^21^,^22^. Therefore, an increased amount of hydrogen generated from the dissociation of ammonia may be the main reason for the lower indium content of InGaN MQWs when the NH_3_ partial pressure is larger.

**Figure 4:**
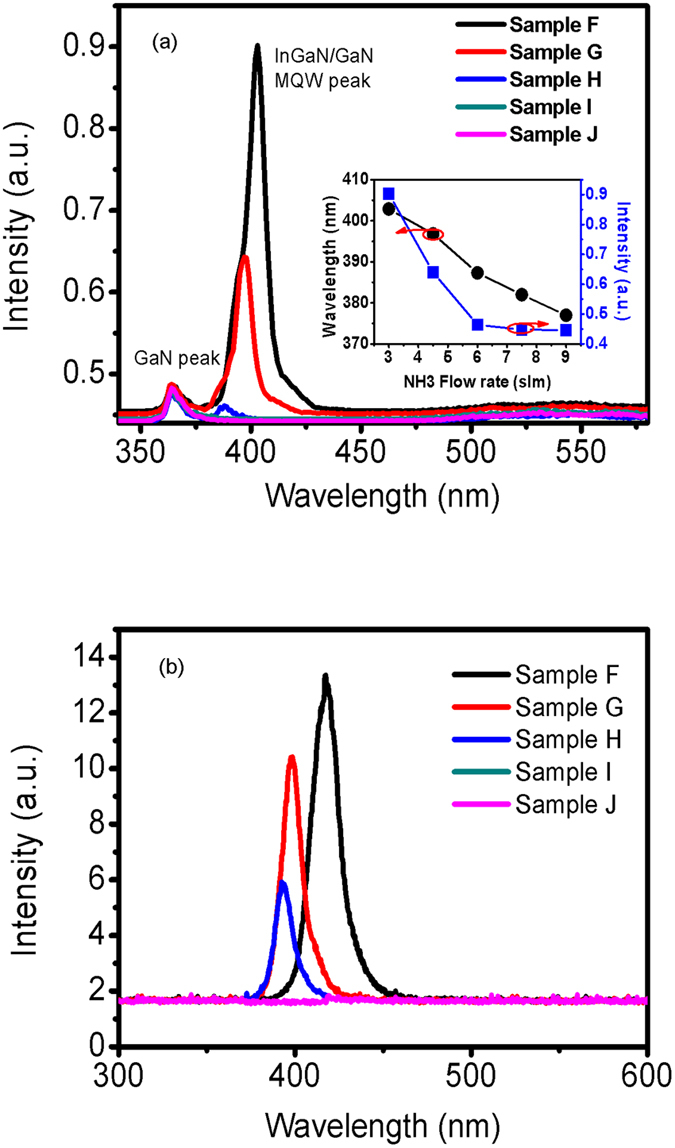
RT PL (**a**) and EL (**b**) of samples F, G, H, I and J. the inset is the dependence of wavelength and peak intensity on the NH_3_ flow rate during InGaN/GaN MQW growth.

**Figure 5:**
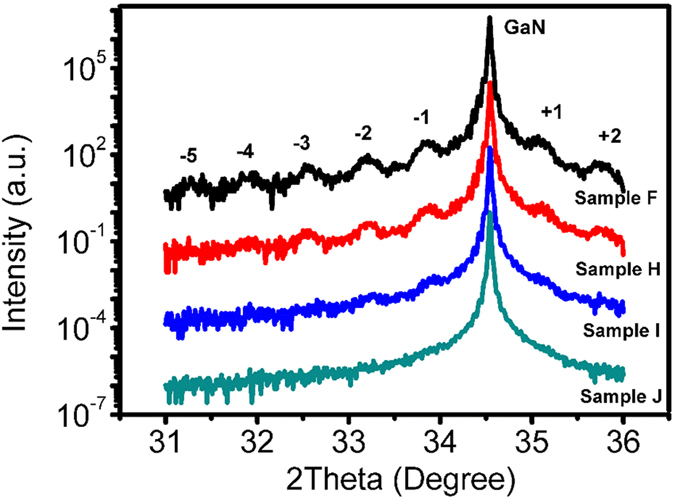
XRD Omega-2theta curves on GaN (0002) plan for Samples F (black), H (red) I (blue) and J (green).

**Figure 6:**
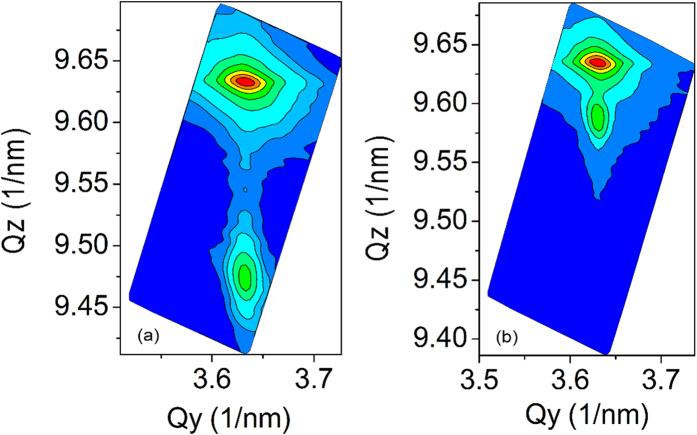
The asymmetrical (10–15) XRD RSM of samples T1 (**a**) and T2 (**b**).

In order to further check this viewpoint and examine the influence of increased growth temperature on the dissociation process of NH_3_, two additional series of samples (F, G, H, I, J of series II and T1, T2 of series III) are grown at a higher temperature of 760 °C. The detail growth conditions are listed in Table 1, then RT PL, EL and XRD Omega-2theta scan of the samples F, G, H, I, and J are measured. The results are shown in Figs 4 and 5. The indium content of InGaN QWs obtained from XRD data for series II are also list in Table 1. It is interesting to note that from both PL and EL spectra the peak intensity and wavelength of InGaN/GaN MQWs decreases abruptly when the NH_3_ flow rate increases from 3 to 6 slm, and the emission peak of InGaN/GaN MQWs nearly disappears when the NH_3_ flow rate increases up to above 7.5 slm (samples I and J). Such a result implies that the rise of growth temperature may much enhance the effect induced by the NH_3_ dissociation on the luminescence of InGaN/GaN MQWs. In addition, it can be seen from Fig. 5 that the 0-th and high order satellite peaks for InGaN/GaN MQWs I and J become very difficult to detect, and the Omega-2theta curve of samples J is quite symmetric in the range between 34 and 35°, similar to that of a n-GaN layer, indicating that the indium content in this sample is very low. Due to the 0-th satellite peak of InGaN/GaN MQWs is too weak to recognize, in order to accurately check the indium content in InGaN/GaN MQWs grown with either high or low NH_3_ flow rate, two additional InGaN films T1 and T2 of series III are grown, their growth conditions are the same as those of InGaN QWs in samples F and J, respectively. The reciprocal space maps (RSMs) of samples T1 and T2 of (10–15) reflection are studied, as shown in Fig. 6. It can be seen that the reciprocal lattice points (RLPs) of InGaN layer and GaN buffer layer have the same Q_y_ for samples T1 and T2, indicating that the InGaN layer is grown coherently on the GaN buffer. However, the Q_z_ value of InGaN layer for sample T1 is much smaller than that of sample T2, indicating that the indium content in InGaN layer with lower NH_3_ flow rate is much higher than that with higher NH_3_ flow rate. It means that a strong corrosive effect of NH_3_ indeed exists during InGaN growth and it will be severely enhanced at a higher NH_3_ partial pressure, especially when a higher growth temperature is taken.

It is known that indium metal droplets are easy to form on the growing surface when the NH_3_ partial pressure is too low, and in this case the decomposition of InGaN layer occurs. In our two series of MQW samples, the diffraction peak related to indium metal is only observed in sample A (grown with low NH_3_ flow rate and low growth temperature) but not found in samples I and J (grown with high NH_3_ flow rate). It indicates that, different from the case of low NH_3_ flow rate, the low indium content in samples I and J can only be attributed to that the chemical reaction for InN growth is suppressed by hydrogen, but has nothing to do with In droplet’s formation. When the NH_3_ partial pressure is too large, most of indium atoms are desorbed from the growing surface of InGaN and are carried away from the reactor by air flow, leading to a low indium incorporation efficiency for InGaN growth. Thus the suppression of the dissociation of NH_3_ during InGaN growth is a very important point for obtaining high indium content InGaN/GaN MQWs with a high quality.

## Conclusion

Three series of samples are grown by MOCVD to investigate the effect of NH_3_ flow rate on optical and structural properties of InGaN/GaN MQWs. It is found that the indium content and emission intensity of InGaN/GaN MQWs depends on the NH_3_ flux and growth temperature. They will decrease when the NH_3_ partial pressure is too large or too small. Different from the case of low NH_3_ flow rate, the corrosive effect of NH_3_ may play a more important role during InGaN growth when NH_3_ partial pressure is large, especially at a relatively high growth temperature. The hydrogen generated from the dissociation of NH_3_ has a corrosive effect to prevent from the chemical reaction between In and N atoms, leading to a low indium content in InGaN/GaN MQWs. It may also result in a more shallow localization states, and therefore a lower emission intensity.

## Additional Information

**How to cite this article**: Yang, J. *et al*. Investigation on the corrosive effect of NH_3_ during InGaN/GaN multi-quantum well growth in light emitting diodes. *Sci. Rep.* **7**, 44850; doi: 10.1038/srep44850 (2017).

**Publisher's note:** Springer Nature remains neutral with regard to jurisdictional claims in published maps and institutional affiliations.

## Figures and Tables

**Table 1:** Growth temperature, TMIn/(TMIn+TMGa), NH_3_ flow rate, total flow in reactor, period thickness of InGaN/GaN MQWs and indium content of InGaN QWs for all samples (-: The XRD satellite peaks of two samples are not observed).

Series	Sample	Temperature (°C)	TMIn/(TMIn+TMGa)	NH_3_ (slm)	Total flow (slm)	Period thickness(nm)	In content (%)
I	A	720	40.6%	0.75	11	13.89	14.9
	B	720	40.6%	1.5	11		
	C	720	40.6%	3	11	13.89	16.2
	D	720	40.6%	4.5	11	14.27	16.1
	E	720	40.6%	6	11	14.3	13.4
II	F	760	40.6%	3	11	14.3	13.8
	G	760	40.6%	4.5	11	14.0	11.2
	H	760	40.6%	6	11	14.0	9.3
	I	760	40.6%	7.5	11	—	—
	J	760	40.6%	9	11	—	—
III	T1	760	40.6%	3	11	46	15.2
	T2	760	40.6%	9	11	49	4.5

**Table 2:** Parameters (ΔE, σ, *IQE*) of samples A, B, C, D and E, which are obtained from temperature dependent PL measurement.

Sample	A	B	C	D	E
ΔE (meV)	2.3	4	7	5.6	3.8
σ (meV)	8.5	12.37	15.65	12.96	10.2
IQE (%)	3.3	7.6	21.5	19.4	6.1
